# The Evolution of Feeding Mechanics in the Danioninae, or Why Giant Danios Don't Suck Like Zebrafish

**DOI:** 10.1093/iob/obac049

**Published:** 2022-12-02

**Authors:** M R Conith, D Ringo, A J Conith, A Deleon, M Wagner, S McMenamin, C Cason, W J Cooper

**Affiliations:** Department of Biology, Western Washington University, Bellingham, WA 98225, USA; Department of Biology, Western Washington University, Bellingham, WA 98225, USA; Department of Biology, University of Massachusetts Amherst, Amherst, MA 01003, USA; School of Biological Sciences, Washington State University, Pullman, WA 99164, USA; School of Biological Sciences, Washington State University, Pullman, WA 99164, USA; Biology Department, Boston College, Chestnut Hill, MA 02467, USA; Marine and Coastal Science, Western Washington University, Bellingham, WA 98225, USA; Department of Biology, Western Washington University, Bellingham, WA 98225, USA; Marine and Coastal Science, Western Washington University, Bellingham, WA 98225, USA

## Abstract

By linking anatomical structure to mechanical performance we can improve our understanding of how selection shapes morphology. Here we examined the functional morphology of feeding in fishes of the subfamily Danioninae (order Cypriniformes) to determine aspects of cranial evolution connected with their trophic diversification. The Danioninae comprise three major lineages and each employs a different feeding strategy. We gathered data on skull form and function from species in each clade, then assessed their evolutionary dynamics using phylogenetic-comparative methods. Differences between clades are strongly associated with differences in jaw protrusion. The paedomorphic *Danionella* clade does not use jaw protrusion at all, members of the *Danio* clade use jaw protrusion for suction production and prey capture, and members of the sister clade to *Danio* (e.g., *Devario* and *Microdevario*) use jaw protrusion to retain prey after capture. The shape of the premaxillary bone is a major determinant of protrusion ability, and premaxilla morphology in each of these lineages is consistent with their protrusion strategies. Premaxilla shapes have evolved rapidly, which indicates that they have been subjected to strong selection. We compared premaxilla development in giant danio (*Devario aequipinnatus*) and zebrafish (*Danio rerio*) and discuss a developmental mechanism that could shift danionine fishes between the feeding strategies employed by these species and their respective clades. We also identified a highly integrated evolutionary module that has been an important factor in the evolution of trophic mechanics within the Danioninae.

## Russian Abstract

Связав анатомическую структуру с механическими характеристиками, мы можем улучшить наше понимание того, как отбор формирует морфологию. В настоящей работе мы рассмотрели функциональную морфологию питания рыб подсемейства Danioninae (отряд Cypriniformes) для определения аспектов краниальной эволюции, связанных с их трофической диверсификацией. Данионины состоят из трех основных линий, каждая из которых использует свою стратегию питания. Мы собрали данные о форме и функциях черепа у видов в каждой кладе, а затем оценили их эволюционную динамику, используя филогенетически-сравнительные методы. Различия между кладами тесно связаны с различиями в выступании челюстей. Педоморфная клада *Danionella* вообще не использует выпячивание челюстей, члены клады *Danio* используют выпячивание челюстей для всасывания и захвата добычи, а представители сестринской клады *Danionella* (например, *Devario* и *Microdevario*) используют выпячивание челюстей для удержания добычи после поимки. Форма предчелюстной кости является основным фактором, определяющим способность к протрузии, и морфология предчелюстной кости в каждой из этих ветвей согласуется с их стратегиями протрузии. Предчелюстные формы быстро эволюционировали, что указывает на то, что они подверглись сильному отбору. Мы сравнили развитие предчелюстной кости у гигантского данио (*Devario aequipinnatus*) и рыбок данио (*Danio rerio*) и обсудили механизм развития, который мог бы смещать рыб-данионинов между стратегиями питания, используемыми этими видами, и их соответствующими ветвями. Мы также определили высоко интегрированный эволюционный модуль, который был важным фактором в эволюции трофической механики у Danioninae.

## Introduction

The form-to-function connection links the developmental processes of morphogenesis to ecological processes that determine fitness. Anatomical structures that shape aspects of mechanical performance critical to niche determination are important targets for evolutionary–developmental (Evo–Devo) study, and their identification can be facilitated by phylogenetic-comparative analyses of functional morphology (Cooper et al. 2017, 2020). Once identified, these features may be targeted for experimental work directed at understanding how ontogenetic changes have shaped patterns of adaptive diversification. If phylogenetic-comparative studies include model species, then the feasibility of performing developmental investigations relevant to understanding lineage evolution is greatly enhanced (Parichy 2006).

The zebrafish (*Danio rerio*, Hamilton 1822; Danionidae, Danioninae) and its close relatives provide an opportunity to gain insight into the evolution and development of fish feeding. Our understanding of zebrafish skull morphogenesis is extensive (e.g., Neuhauss et al. 1996; Schilling et al. 1996; Wada et al. 2005; Nguyen et al. 2022), the array of investigative tools available for this species can be adapted for use with closely related fishes (Parichy and Johnson 2001; Parichy 2006; McMenamin et al. 2014), and work on zebrafish feeding mechanics provides a foundation for comparative studies of trophic diversification in its lineage (Hernandez 2000; Hernandez et al. 2007; Staab and Hernandez 2010; Tang et al. 2010; McCluskey and Postlethwait 2015; Stout et al. 2016; Galindo et al. 2019; Keer et al. 2019). Nonetheless, no previous studies have examined feeding mechanics in more than two danionid fishes. Here we investigated cranial form and function in the subfamily Danioninae (*sensu*Stout et al. 2016) and analyzed data using phylogenetic-comparative methods. We also compared aspects of trophic development in two species with divergent feeding strategies: zebrafish and giant danio (*Devario aequipinnatus*; McClelland 1839).

The Danioninae (*sensu*Stout et al. 2016) comprise 101 known species that inhabit a range of freshwater habitats in South and Southeast Asia (Talwar and Jhingran 1991; Fang et al. 2009; Tang et al. 2010). Their evolutionary relationships have been the subject of considerable study (e.g., Fang 2003; Mayden et al. 2007; Fang et al. 2009; Tang et al. 2010; Liao et al. 2011; McCluskey and Postlethwait 2015) and extensive, well-supported phylogenies are now available (Tang et al. 2010; Stout et al. 2016). The most recent of these restricts the Danioninae to the following nine genera distributed among three clades: *Chela, Danio, Danionella, Devario, Inlecypris, Laubuka, Microdevario, Microraasbora*, and *Neochela* (Stout et al. 2016). The genus *Danionella* constitutes the most basal clade, and the genus *Danio* is the sister lineage to the remaining taxa (Stout et al. 2016). We sampled from all three clades and examined species whose genera account for approximately 80% of danionine species diversity (Stout et al. 2016).

**Fig. 1:**
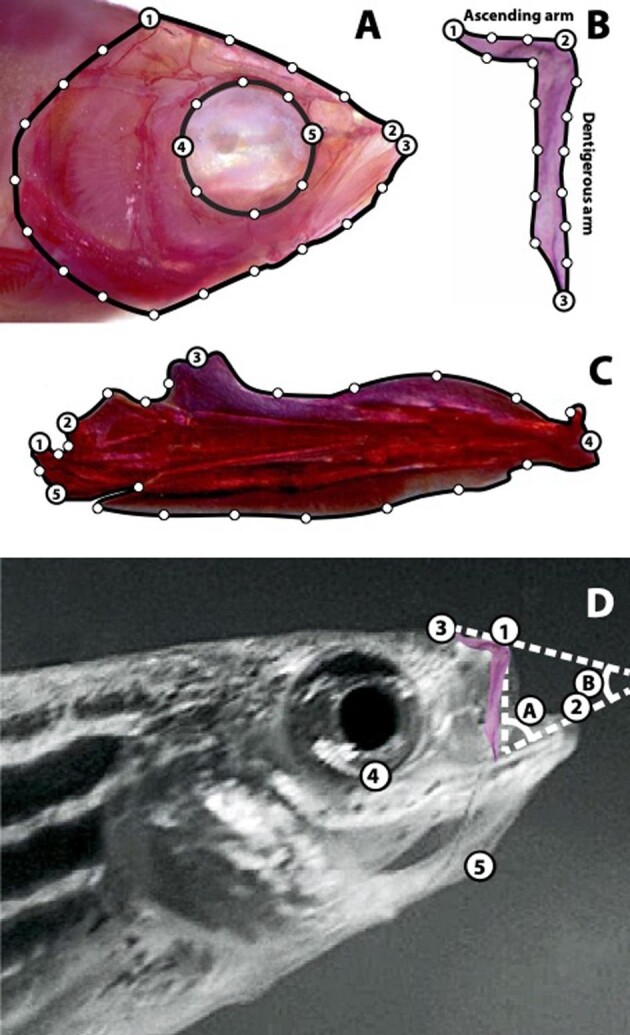
Shape and motion data collection. A. Homologous anatomical landmarks (LM) used in skull shape analyses: (1) most posterio–dorsal point on the parietal bone; (2) anterior tip of the premaxilla; (3) anterior tip of the dentary; (4) posterior point on the eye socket in line with the parasphenoid; and (5) anterior point on the eye socket in line with the parasphenoid. Semilandmarks were evenly spaced along the outline of the skull between LM 1 and 2, 1 and 3, 4 and 5, and 5 and 4. B. Homologous anatomical LM used in premaxilla shape analyses: (1) tip of the ascending arm; (2) anterior tip of the premaxilla (see panel D for the location of the premaxilla within the skull); (3) and tip of the dentigerous arm. Semilandmarks were evenly spaced along the outline of the premaxilla between LM 1 and 2, 2 and 3, and 1 and 3. C. Homologous anatomical LM used in mandible shape analyses: (1) posterio–dorsal extent of the lower jaw joint (quadrate–articular joint); (2) anterio–dorsal extent of the lower jaw joint; (3) tip of the primordial process of the articular; (4) anterior tip of the dentary; and (5) posterior tip of the angular. Semilandmarks were evenly spaced along the outline of the mandible between LM 1 and 2, 2 and 3, 3 and 4, 4 and 5, and 1 and 5. D. LM and angles used in motion analyses: (1) anterior tip of the premaxilla; (2) anterior tip of the dentary; (3) a stationary point on the dorsal surface of the anterior head; (4) ventral-most point of the eye socked; (5) anterior tip of the hyoid series (basihyal); (A) gape angle; and (B) angle of jaw-protrusion direction.

Jaw protrusion is a key innovation that likely contributed to the considerable success of the Cypriniformes (∼3200 species) and other fish lineages in which the ability has evolved convergently (e.g., Acanthomorpha, ∼17,000 species; Wainwright et al. 2015). Differences in jaw-protrusion mechanics have been tightly linked to diet in other fishes (Cooper et al. 2017), suction production via jaw protrusion is an important component of zebrafish feeding (Fig. 1; Hernández et al. 2002; Staab et al. 2012; Hernandez and Staab 2015), and highly mobile upper jaw elements are a synapomorphy of the cypriniform order to which the Danioninae belong (Staab et al. 2012; Wainwright et al. 2015). We examined multiple aspects of danionine trophic form and function (Table 1), with a particular emphasis on the timing, extent, direction, and anatomical determinants of upper jaw protrusion.

**Table 1:** Anatomical and motion variables analyzed.

Variable	Description when not self-explanatory
(1) Eye diameter	
(2) Mandible length	
(3) Premaxilla ascending arm length	
(4) Premaxilla ascending arm length/premaxilla dentigerous arm length	
(5) Premaxilla ascending arm length/mandible length	
(6) Mandible opening mechanical advantage (MA)	See text
(7) Mandible closing MA	See text
(8) Maximum jaw-protrusion distance	
(9) Jaw-protrusion angle at maximum protrusion	See Fig. 1D
(10) Jaw-protrusion speed	Speed calculated as proportion of body length/time (mm/SL/ms)
(11) Jaw-protrusion speed (mm/ms)	Speed in mm/s; not standardized by fish SL
(12) Time from strike onset to the onset of jaw protrusion	
(13) Time from strike onset to maximum jaw protrusion	
(14) Time from the onset of jaw protrusion to prey capture	
(15) Time from the onset of jaw protrusion to maximum jaw protrusion	
(16) Time from maximum jaw protrusion to prey capture	
(17) Gape angle change at maximum jaw protrusion	Some fishes maintained fully protruded jaws as the mouth closed
(18) Gape angle at maximum jaw protrusion	Gape angle when max jaw protrusion was first reached
(19) Time from the onset of jaw protrusion to maximum gape	
(20) Time from maximum jaw protrusion to maximum gape	
(21) Time from maximum hyoid depression to the onset of jaw protrusion	
(22) Time from maximum hyoid depression to maximum jaw protrusion	
(23) Maximum gape distance	
(24) Maximum gape angle	
(25) Gape speed	Speed calculated as proportion of body length traveled/time (mm/SL/ms)
(26) Gape speed (mm/ms)	Speed in mm/s; not standardized by fish SL
(27) Maximum hyoid depression distance	
(28) Hyoid depression speed	Speed calculated as proportion of body length traveled/time (mm/SL/ms)
(29) Hyoid depression speed (mm/ms)	Speed in mm/s; not standardized by fish SL
(30) Strike duration	
(31) Strike distance	Distance from fish to prey at strike onset
(32) Strike distance (mm)	Distance not standardized by fish SL
(33) Distance traveled from strike onset to prey capture	Strike distance minus the distance prey were pulled toward a fish via suction
(34) Strike speed	Speed calculated as proportion of body length traveled/time (mm/SL/ms)
(35) Strike speed (mm/ms)	Speed in mm/s; not standardized by fish SL
(36) Time from strike onset to prey capture	

Standard length (SL), distances in mm/SL unless stated otherwise, angles in degrees, time in milliseconds (ms).

The goals of this study were to describe patterns of evolutionary divergence in the functional morphology of feeding in the Danioninae, and to identify aspects of their cranial shape that distinguish between alternate feeding strategies. The large number of genetically modified zebrafish lines that exhibit changes in craniofacial development (Neuhauss et al. 1996; Yelick and Schilling 2002; Parsons et al. 2011), the range of investigative tools that have been developed for working with this species (Kimmel et al. 1995; Thisse and Thisse 2005, 2008; Hwang et al. 2013), and the ease with which many of its closest relatives can be reared in captivity (Parichy and Johnson 2001; Parichy 2006; McMenamin et al. 2014) make this lineage highly amenable to comparative studies of skull morphogenesis. The relevance of such work to understanding their adaptive diversification is enhanced by knowing which cranial features are associated with ecologically significant aspects of functional performance. By describing evolutionary patterns in their cranial biomechanics, this project will promote our ability to identify the developmental changes that have shaped the diversification of feeding strategies in the Danioninae.

## Materials and methods

### Specimen acquisition, rearing, and breeding

Zebrafish specimens (wild-type, AB line) were produced by natural matings. Specimens of *Danio albolineatus* (Blyth 1860), *Danio erythromicron* (Annandale 1918), *Danio feegradei* (Hora 1937), *Danio quagga* (Kullander et al. 2009), *Danionella dracula* (Britz et al. 2009), *Devario aequipinnatus, Devario maetaengensis* (Fang 1997), and *Microdevario kubotai* (Kottelat and Witte 1999) were obtained from the pet trade (The Wet Spot Tropical Fish, Portland, OR, USA). These species represent all three of the major branches of the Danioninae (*sensu*Stout et al. 2016): (1) *Danionella* (basal), (2) *Danio*, and (3) the sister clade to *Danio* (this branch includes *Devario* and *Microdevario* [DM] and will be referred to as DM henceforward).

All fishes were reared and/or maintained in standard zebrafish housing (Aquaneering, San Diego, CA, USA) except for adult *Devario aequipinnatus*, which were kept in 20 gallon glass aquaria with circulating water filtered through a fluidized bed (Aquaneering). Environmental conditions for all species were identical to those used to maintain zebrafish: all fishes—14/10 light/dark cycle, 28°C, pH ∼7.0 (6.8–7.5); embryos/young larvae (0–5 days post fertilization; dpf)—kept in petri dishes in an incubator until capable of feeding; larvae (5–20 dpf)—maintained in 2.5 L tanks in an incubator, initially fed live *Paramecia* and transitioned to live, newly hatched (nauplius stage) brine shrimp (*Artemia* sp.); older larvae/juveniles (20 dpf onward)—maintained in 2.5 or 9.0 L tanks on a recirculating zebrafish system (Aquaneering) with low water flow that was increased gradually, fed a diet of live *Artemia* to which finely ground flake food was slowly introduced; adults—maintained in 9.0 L tanks on a recirculating zebrafish system, fed tropical fish flake food and/or live *Artemia* twice per day. All fish care and euthanasia followed approved Washington State University (where data collection occurred) animal care protocol 4285.

Zebrafish were bred by placing 4–6 male/female pairs in 1.7 L sloped breeding tanks (Techniplast, West Chester, PA, USA) overnight and collecting eggs the following morning. *Devario aequipinnatus* (giant danio) were induced to breed by reducing their water temperature by ∼3–5°C after females appeared gravid. A glass tray topped with short, artificial aquarium plants was placed in the center of the aquarium as a breeding location. *Devario aequipinnatus* predominantly bred above and among these plants soon after lights came on the morning following a water temperature reduction. Their eggs, which are slightly adhesive, were collected and their embryos, larvae, and juveniles were raised using a zebrafish protocol.

### High-speed video analyses

All adult fishes were acclimated to glass aquaria and filmed feeding on live *Artemia*. Fishes were filmed in lateral view at 500 frames per second using an Edgertronic monochrome high-speed video camera (Sanstreak Corp., San Jose, CA, USA). We selected two to four clear strike sequences for each of two to four individuals per species. The ImageJ software program (Schneider et al. 2012) was used to measure distances and angles on images extracted from these videos and to track changes in these measurements over the course of a feeding strike. Strike sequences were considered to begin at the onset of mouth opening and to end when the mouth had fully closed, and both the premaxilla and the hyoid had also been fully adducted and thus returned to the pre-strike state. For each sequence the frame in which an *Artemia* nauplius first disappeared into the mouth established the time of prey capture.

On every video frame we measured the following five variables (see Fig. 1D for reference): (1) Jaw-protrusion distance (the distance between points 1 and 3, minus the original distance between these points before jaw protrusion began; (2) Gape distance (the distance between points 1 and 2); (3) Hyoid depression distance (the distance between points 4 and 5, minus the original distance between these points before hyoid depression began); (4) Gape angle (angle A); and (5) Jaw-protrusion angle (angle B). Determining the onset times for jaw protrusion, gape, and hyoid depression, as well as the time at which each of these motions reached their maximum and the time of prey capture, allowed us to quantify the relative timing of a large number of variables. We also calculated the distance between fishes and their prey at strike onset, the distance traveled over the course of a feeding strike, and the average strike speed. In total, we measured 29 variables that described the extent and timing of motions that accompanied feeding in these fishes (variables 8–36 in Table 1).

### Shape analyses

The specimens used for the collection of video data were euthanized, formalin fixed until rigid, leached of formalin in tap water, and stepped into 70% ethanol for storage. Preserved specimens were cleared and stained (Potthoff 1984) before digital imaging. Images of heads were taken in lateral view with the mouth closed. Premaxillae and mandibles were then removed and photographed in lateral view. All images were collected using an Olympus DP25 digital camera interfaced with an Olympus SZ61 stereomicroscope. It should be noted that there is some debate as to whether the highly paedomorphic *Danionella* possess premaxillae at all, or instead have only maxillae in their upper jaws (Britz et al. 2009). Although we included their upper jaw elements in our premaxillary shape analyses, these structures may not be truly homologous with the premaxillae of other species. The upper jaw elements of male *Danionella dracula* are highly derived, presumably via sexual selection, and they possess large, fang-like, bony processes that are apparently not used for feeding (Britz et al. 2009). Because of this extreme dimorphism, only upper jaw elements from females were included in the premaxillary shape analyses, and only female *D. dracula* were filmed during feeding. Early juvenile zebrafish and *Devario aequipinnatus* were euthanized during the initial stages of upper jaw ossification. After clearing and staining the premaxillae were removed from these specimens for imaging.

Geometric morphometrics were used to quantify the lateral shapes of skulls, premaxillae and mandibles (Fig. 1A–C). Analyses were based on species shape averages calculated from all specimens used for video analysis. The StereoMorph package (Olsen and Westneat 2015), which runs in the R programming environment (R Core Team 2020), was used to quantify anatomical shape by determining the coordinate locations of homologous anatomical landmarks (LM) and semilandmarks on images of skulls, premaxillae, and mandibles (Fig. 1). We used the geomorph package in R (Baken et al. 2021) to perform a generalized Procrustes analysis (GPA) to remove the effects of size, translation, and rotation from each suite of landmark configurations via the gpagen function.

We used a phylomorphospace analysis (PA) to visualize shape in a phylogenetic context (Rohlf 2002; Sidlauskas 2008). A standard principal component analysis was performed to construct a morphospace along major axes of shape variation and their phylogeny was projected onto this morphospace. Complementary to this analysis, we performed a phylogenetically aligned component analysis (PACA) to visualize the morphological variation most aligned with phylogenetic signal (Collyer and Adams 2021a). Comparing a PACA plot with a PA plot reveals whether the primary axis of shape variation in the data is due to phylogeny or other factors such as ecology. For both PA and PACA we used the gm.prcomp function in the geomorph package in R (R Core Team 2020; Adams et al. 2021; Baken et al. 2021).

StereoMorph was also used to measure eye diameter, premaxilla ascending arm length, premaxilla dentigerous arm length, mandible length, the distance from the lower jaw joint to the dorsal tip of the primordial process of the mandible (mandible closing in-lever length; see following text), and the distance from the lower jaw joint to the posterio–ventral tip of the angular bone (mandible opening in-lever length; see see following text).

The mandible represents a lever system in which: (1) muscular force applied to the angular bone contributes to mouth opening; (2) muscular force applied to the primordial process contributes to mouth closing; and (3) the lower jaw joint acts as a fulcrum for both motions (Westneat 1994). The length of the mandible closing in-lever relative to total mandible length determines the mechanical advantage (MA) applied to this system during mouth closing. Likewise, the length of the mandible opening in-lever relative to total mandible length determines the MA applied to this system during mouth opening.

### Evolutionary analyses

We used a pruned maximum likelihood (ML) tree from Tang et al. (2010) because it is extensive, well-resolved, and the only published phylogeny that includes all 9 of the species in our dataset. We chose to use the ML tree instead of the parsimony tree presented in the main text of Tang et al. (2010) because the topology of the ML tree is more consistent with the most recent work on danionine phylogenetics (McCluskey and Postlethwait 2015; Stout et al. 2016). We used a penalized likelihood method contained in the chronos function from the R package ape to transform this tree into an ultrametric version (Paradis et al. 2004). The chronos function uses a penalized likelihood method to convert branch lengths from the number of substitutions per site to time. We time-calibrated the branches using a fossil representative of the most recent common ancestor of *Danio* and *Cyprinus*, and set a soft bound estimated to 49–140.2 Ma (Kappas et al. 2016).

Differences in the shapes of the skull, premaxilla, and mandible between clades were assessed with a Procrustes ANOVA using the procD.lm function in the geomorph package in R. The clades in question are the three major divisions of the Danioninae *sensu*Stout et al. (2016) that are represented in this study by specimens of *Danionella, Danio*, and DM, respectively. We maximized statistical power for factorial models by using the randomized residual permutation procedure (RRPP; Collyer and Adams 2018, 2021b). For each shape dataset, size was included in the model if it was significant in a model selection step. Size was a significant factor for both the mandible (*P* = 0.04) and head (*P* = 0.01) datasets; therefore, the final model included both size and clade (shape ∼ log[centroid size] + clade). However, for the premaxilla dataset, size was not significant (*P* = 0.95) and it was therefore excluded from the final model (shape ∼ clade). When clade was a significant factor we performed pairwise comparisons using the pairwise function in the RRPP package in R (Collyer and Adams 2018, 2021b) to determine which clades were driving this pattern.

We used phylogenetic ANOVA (pANOVA) to determine if there is a relationship between our kinematic variables and aspects of danionine ecology. The phenotypic characters we measured are most relevant to diet, but only limited diet data are available for danionines (McClure et al. 2006; Spence et al. 2007). As an alternative we used published records of habitat and maximum body size as the grouping variables in our model (Pantulu 1986; Talwar and Jhingran 1991; Fang 1998; McClure et al. 2006; Spence et al. 2007, 2008; Britz et al. 2009; Kullander and Britz 2015; Parichy 2015; Froese and Pauly 2022). Body size can be an important determinant of which food sources a species can utilize (Pessanha and Araujo 2014; Guedes et al. 2015) and the physical environments of disparate habitats can require the use of different feeding strategies (Higham et al. 2015; Bozeman and Grossman 2019; Pombo-Ayora et al. 2020). We used water speed (fast-moving vs. slow-moving or still) to capture aspects of the environment that might influence feeding kinematics (Higham et al. 2015; Bozeman and Grossman 2019). Since body size is not a discrete variable, we used three different size groupings to be sure patterns weren't being driven by a certain size cutoff (Table 2). Details of these groupings can be found in Table 2.

**Table 2:** Phylogenetic ANOVA species groupings and test results.

		Phylogenetic ANOVA groupings			
Species	Maximum SL (mm)	Size 1	Size 2	Size 3	Environment
*Danionella dracula*	17.0	Small	Small	Small	Slow/still water
*Danio albolineatus*	65.0	Medium	Large	Medium	Fast water
*Danio erythromicron*	30.0	Small	Medium	Medium	Slow/still water
*Danio feegradei*	68.0	Medium	Large	Medium	Slow/still water
*Danio quagga*	45.0	Medium	Medium	Medium	Fast water
*Danio rerio* (zebrafish)	38.0	Medium	Medium	Medium	Slow/still water
*Devario aequipinnatus* (giant danio)	150.0	Large	Extra large	Large	Fast water
*Devario maetaengensis*	50.0	Medium	Medium	Medium	Fast water
*Microdevario kubotai*	19.0	Small	Small	Small	Slow/still water
*P*-values (above) and *F*-statistics (below) for significant phylogenetic ANOVA results					
	Mandible	Distance traveled from		Maximum gape	
Grouping	length	strike onset to prey capture	Strike speed	distance	Gape speed
Size 1	0.011	0.036	0.016		0.017
	17.675	6.622	7.889		12.279
Size 2	0.008	0.028	0.036	0.028	0.049
	19.604	6.973	5.499	10.317	6.421
Size 3	0.03	0.034	0.035	0.044	
	12.744	7.366	6.487	12.334	
*P*-values for pairwise phylogenetic ANOVA comparisons					
	Mandible	Distance traveled from strike		Maximum gape	
	length	onset to prey capture	Strike speed	distance	Gape speed
Size 1					
L:M	0.005*	0.004*	0.005*	NA	0.003*
L:S	0.070	0.088	0.116	NA	0.118
M:S	0.613	0.609	0.489	NA	0.324
Size 2					
XL:L	0.002*	0.107	0.002*	0.054	0.002*
XL:M	0.035*	0.002*	0.023*	0.098	0.037*
XL:S	0.054	0.214	0.121	0.004*	0.050
L:M	0.332	0.363	0.409	0.539	0.299
L:S	0.598	0.805	0.45	0.623	0.731
M:S	0.974	0.446	0.708	0.358	0.862
Size 3					
L:M	0.019*	0.004*	0.013*	0.098	NA
L:S	0.064	0.191	0.131	0.006*	NA
M:S	0.845	0.521	0.625	0.402	NA

Environment: no significant results. Significant pairwise comparisons indicated in bold with an asterisk.

We performed pANOVA on all kinematic variables for which we had data for all nine species (*n* = 17). Because there is no jaw protrusion in *Danionella* (McMenamin et al. 2017), we excluded all variables that included jaw protrusion from these analyses. Where appropriate, we used body size- and phylogenetically corrected residuals. A pANOVA was performed using the procD.pgls function from the geomorph package and *post hoc* comparisons were assessed using the pairwise function from the RRPP package in R (Adams et al. 2021; Baken et al. 2021). The pairwise function allowed us to perform *post hoc* pairwise comparisons on significant variables even when there was only one species in a group. This was the case in the body size groupings because *Devario aequipinnatus* was the only species in its group since it is more than twice as long as the next largest species in our dataset (Table 2).

We analyzed the covariance between univariate morphological and kinematic variables while accounting for relatedness using phylogenetic generalized least squares (PGLS) as in Cooper et al. (2017). Briefly, a total of 36 variables (Table 1) were compared using the gls function in the nlme package in R (Pinheiro et al. 2022).

Inspection of video recordings and the results of preliminary analyses indicated that some of the species we examined use jaw protrusion for suction production and prey capture, some species use jaw protrusion for retaining prey after capture, and one species (*Danionella dracula*) did not appear to use jaw protrusion. We therefore compared differences in feeding mechanics between species that use these alternative strategies.

The use of protrusion for prey capture appeared consistent among all *Danio* species, and its use for prey retention appeared consistent among all members of the DM clade. For comparisons in which the independent variable is synonymous with clade, accounting for phylogeny in the error term would be redundant and would result in lower statistical power for detecting existing differences (Adams and Collyer 2018; Dean Adams, personal communication). We therefore used a Student's *t*-test to compare anatomical and motion variables associated with protrusion in the sister clades that do not include *Danionella* (variables 8–21 in Table 1).

We compared rates of premaxillary shape evolution between species that use jaw protrusion either for prey capture or prey retention. Evolutionary rates were assessed under a Brownian motion model using a method that calculates a phylogenetically corrected rate based on a species distance approach (Adams 2014). We first calculated the Brownian rate (σ^2^) of premaxillary shape evolution for each group using our geometric morphometric premaxilla shape data (Fig. 1B) and obtained a ratio of rates between groups. We then determined significance via phylogenetic simulation (10,000 iterations) whereby simulated tip data are obtained under a Brownian motion model of evolution using a common evolutionary rate pattern for all Danioninae species examined. We used the compare.evol.rates function in the geomorph package in R to extract the σ^2^ for each group and determine whether differences in rates were significant (Adams et al. 2021; Baken et al. 2021).

We also compared evolutionary rates for anatomical and motion variables between species that use jaw protrusion for different purposes. To account for uncertainty in discrete character histories, we used the Stochastic Mutational Mapping on Phylogenies (SIMMAP) tool (Bollback 2006). Using the make.simmap function from the phytools package in R (Revell 2012) we generated 500 simulated character history trees using a symmetric rates model that permitted taxa to transition between states at equal rates. We then calculated the σ^2^ of premaxilla ascending arm length and protrusion timing relative to gape for each grouping and assessed significance by comparing the likelihood ratio against a χ^2^ distribution. We used the brownie.lite function (O'Meara et al. 2006) from the phytools package in R (Revell 2012) to conduct Brownian rates analysis, and we report the median values from the simulations.

## Results

### Premaxilla shape and skull shape are distinct among the major danionine clades

**Fig. 2:**
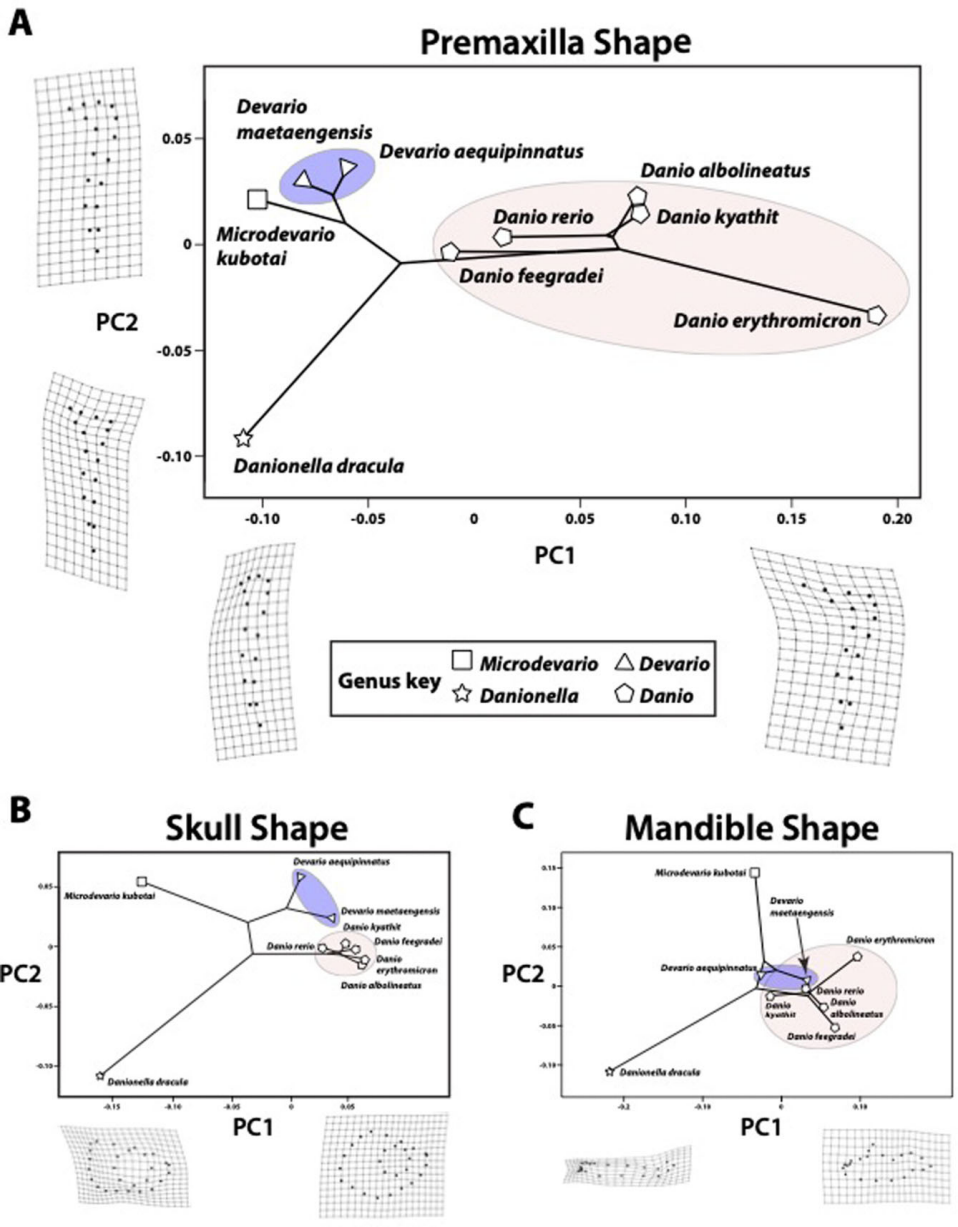
PCA-based phylomorphospaces for premaxilla, skull, and mandible shape diversity in the Danioninae. A. Premaxilla shape diversity. Deformation grids depict the shapes associated with high and low PC scores on both axes. B. Skull shape diversity. Deformation grids depict the shapes associated with high and low PC1 scores. C. Mandibular shape diversity. Deformation grids depict the shapes associated with high and low PC1 scores.

Principal component analyses of coordinate shape data showed that each of the major danionine lineages had premaxilla and skull shapes that were distinct from each other. The upper jaw, skull, and mandible shapes of *Danionella* were the most unique (Fig 2A–C). The skull and mandible shapes of *Microdevario* were likewise very distinct (Fig 2B, C), but the premaxilla shapes of *Microdevario* and *Devario* were similar to each other (Fig. 2A). Skull shape and mandible shape were similar in *Danio* and *Devario*, with some overlap in mandible shape (Fig. 2B, C).

The largest axis of premaxilla shape variation (PC1) was strongly associated with the length of the ascending arm (Figs. 1B and 2A). PC2 was primarily associated with the degree of premaxillary curvature (Fig. 2A). For skull shape, PC1 was strongly associated with skull height, eye size, and the curvature of the posterior margin of the operculum (Fig. 2B). For mandible shape, PC1 was strongly associated with distinguishing the simple, rod-like mandible of *Danionella* from the more complex mandible shapes of the other genera. Species with higher PC1 scores (i.e., all but *Danionella*) had higher primordial processes, more upturned lower-jaw joints, and taller mandibles with greater curvature (Fig. 2C).

Phylogenetic signal accounts for no more than one-third of the variation in shape, according to the results of PACA. The PACA returned a partial RV score for each axis of divergence (called phylogenetically aligned components, PAC), which is analogous to the proportion of variance explained in a standard principal component analysis (PCA) except that it does not sum to 1. The total RV is the sum of all partial RVs and a measure of the covariation between shape and phylogeny. For these data, the total RV for the premaxilla is 33.3%, for the mandible is 21.1%, and for the skull is 26.1%. Most of this variation is seen in PAC1 and in premaxilla, skull, and mandible views, at least 97% of the variation is captured in the first two PACs. The PAC plots (Supplementary Fig. S1) show a broadly similar dispersion across PAC1 when compared to the PC plots (Fig. 2). However, the partial RV score for PAC1 in all views is two to three times lower than the percentage variation explained by PC1 in the standard PCA, which suggests that phylogeny is one of multiple factors that contribute to shape variation.

The results of Procrustes ANOVA tests showed that clade is a significant factor in patterns of shape variation in the premaxilla (*R*^2^ = 0.55, *Z* = 1.83, *P* = 0.03), skull (*R*^2^ = 0.27, *Z* = 1.98, *P* = 0.02), and mandible (*R*^2^ = 0.53, *Z* = 2.86, *P* = 0.002). Pairwise comparisons revealed that the *Danio* clade was distinct from the DM clade in regard to premaxilla (*P* = 0.03) and skull shape (*P* = 0.002). Shape variation between clades in the mandible was driven by the unique anatomy of *Danionella* in comparison to *Danio* (*P* = 0.0005) and to DM (*P* = 0.002).

### Different feeding strategies are employed by each of the major danionine clades

**Fig. 3:**
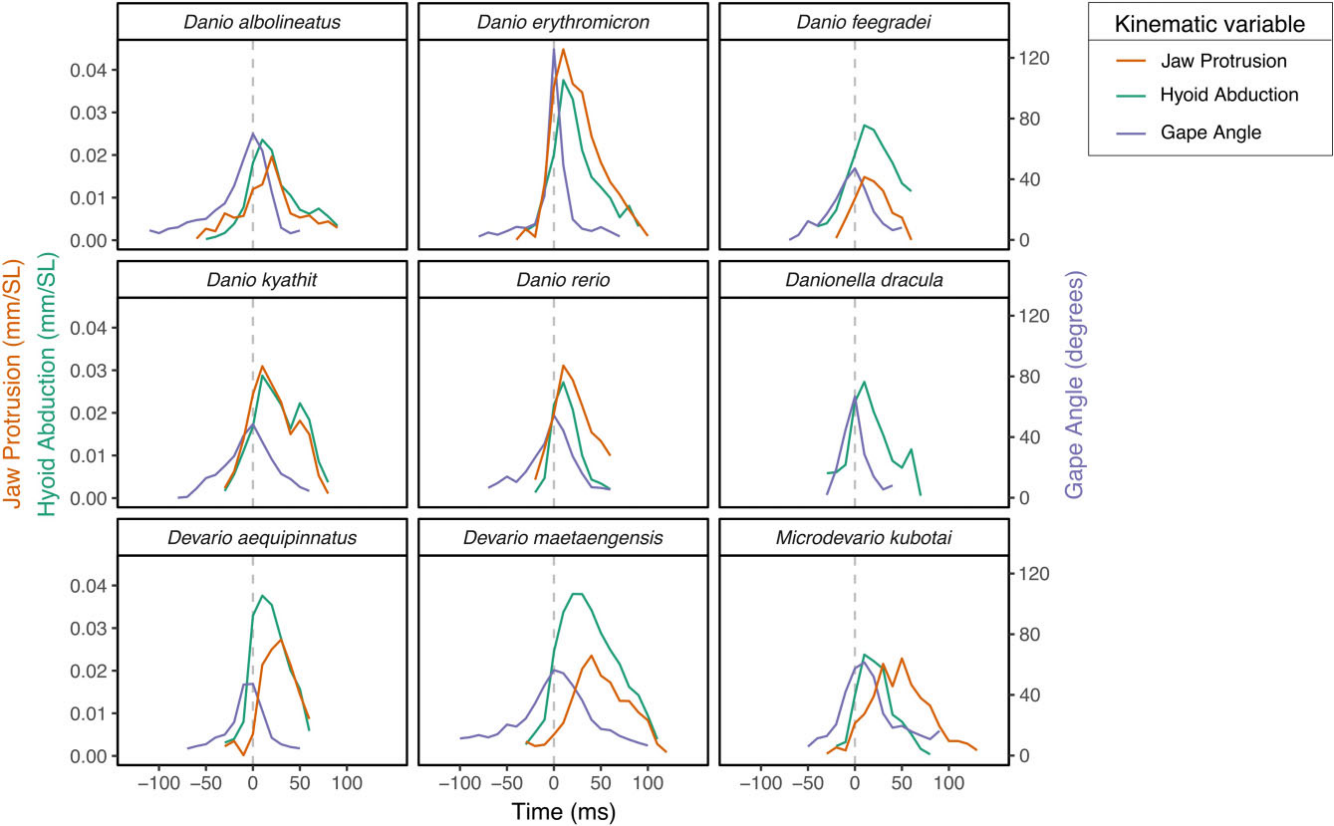
Time series for jaw protrusion, hyoid depression, and gape angle during feeding strikes for nine danionine species. Species means are shown. Horizontal bars indicate the time elapsed between maximum hyoid depression and maximum jaw protrusion. There are no horizontal bars for *Danio erythromicron, D. feegradei, D. quagga*, or *D. rerio* because these maxima occurred simultaneously.

*Danionella* did not exhibit jaw protrusion, but in all other species maximum jaw protrusion occurred after maximum gape and prey capture (Fig. 3). For all species maximum gape was nearly simultaneous with prey capture and both occurred before maximum hyoid depression (Fig. 3). Species that had long ascending arms on their premaxillae (i.e., the *Danio* lineage, see Fig. 2A) exhibited multiple differences in feeding mechanics relative to those that had short ascending arms on their premaxillae (i.e., the DM lineage, see Fig. 2A).

In *Danio* species, maximum jaw protrusion occurred soon after maximum gape (and prey capture), but the time from maximum gape to maximum jaw protrusion was significantly longer in DM (Fig. 3 and Table 3). In comparison to DM species, gape angles were significantly greater in *Danio* at the time of maximum jaw protrusion (Fig. 3 and Table 3). In *Danio*, maximum jaw protrusion and maximum hyoid depression were usually simultaneous or nearly so, with a slight delay in jaw protrusion in *D. albolineatus*, but the time from maximum hyoid depression to maximum jaw protrusion was significantly longer in DM (Fig. 3 and Table 3).

**Table 3:** The *t*-test results.

Significant results of one-tailed *t*-tests that compared species which used jaw protrusion for suction production and prey capture (SP species) to those that used it for prey retention (PR species)				
Variable	Mean SP species	Mean PR species	*t*-value (*df* = 6)	*P*-value
Premaxilla ascending arm length/premaxilla dentigerous arm length*	0.39	0.23	2.0155	0.0452
Jaw-protrusion angle at maximum protrusion*†	39.32	65.70	−3.5980	0.0057
Time from maximum jaw protrusion to prey capture^a^	−12.00	−40.00	5.6125	0.0007
Gape angle at maximum jaw protrusion	48.79	23.73	2.4473	0.0250
Time from maximum jaw protrusion to maximum gape†^a^	−12.00	−36.67	6.8316	0.0002
Time from maximum hyoid depression to maximum jaw protrusion^a^	−2.00	−23.33	3.0604	0.0111

a A negative number indicates that maximum jaw protrusion occurred after prey capture, maximum gape, or maximum hyoid depression.

Angles in degrees, time in milliseconds. Variables that share either of these symbols evolved in a correlated manner (see Table 3): *†

Prey were engulfed by *Danio* species at maximum gape and immediately before jaw protrusion and hyoid depression reached their maxima (Fig. 3). We observed that prey accelerated toward *Danio* species just before prey capture. This acceleration was clearly the result of suction production and not due to prey swimming (e.g., acceleration was often in a different direction than the one in which the prey had been swimming). Maximum hyoid depression in DM also occurred almost immediately after prey capture and maximum gape (Fig. 3), and *Artemia* were also observed to accelerate toward the mouths of these fishes due to suction just before prey capture. Substantial jaw protrusion in DM did not, however, occur until after prey had already been engulfed (Fig. 3). In *Danio* the direction of jaw protrusion was primarily forward and quasi-parallel with the long axis of the mandible, while in DM premaxillary motion was primarily toward the mandible (Table 3).

### Correlated evolution and integration of functional variables

**Fig. 4:**
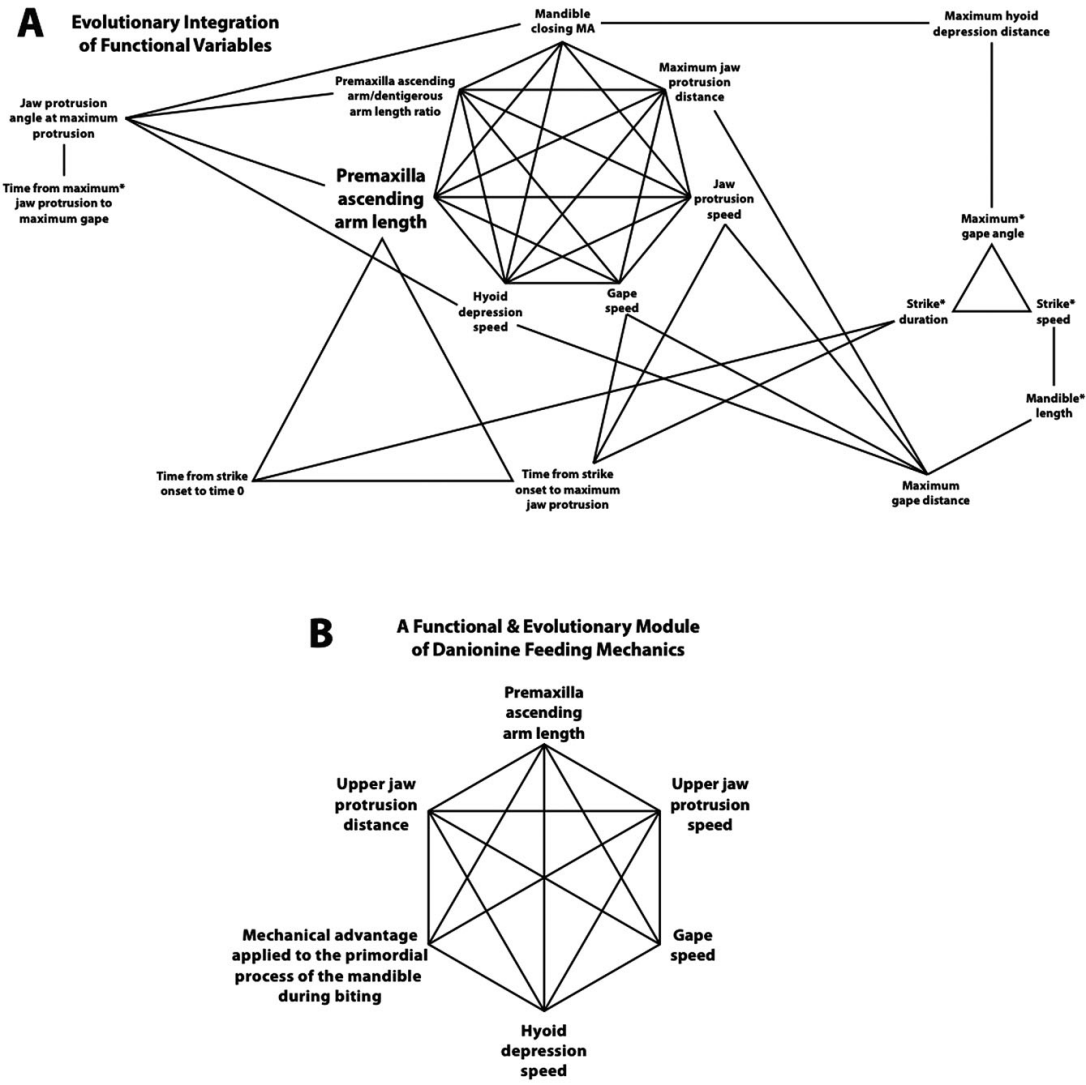
Evolutionary patterns of integration among variables that describe the functional morphology of feeding in the Danioninae. A. Connections between variables indicate that phylogenetic generalized least squares (PGLS) analyses showed their evolution to have been significantly correlated (see Table 4). All correlations were positive. For the five cases in which variables were analyzed in two versions (both raw measurements and measurements adjusted by standard length; see Table 1), only the adjusted measurements were included for the sake of clarity (raw measurements are included in Supplementary Fig. S1). B. A functional and evolutionary module of danionine feeding mechanics. The evolution of these variables has been highly integrated.

Most of the kinematic and morphological variables tested showed a significant correlation with at least one other variable. Only “mandible opening MA” and “Time from maximum jaw protrusion to maximum hyoid depression” did not (Fig. 4, Table 4). Patterns of correlated evolution among groups of variables are contained within tables of PGLS results (e.g., Table 4), but identifying these patterns can be difficult. To aid in visualizing patterns of correlation among variables we provide a network diagram in Fig. 4.

**Table 4:** Results of PGLS tests for correlated evolution.

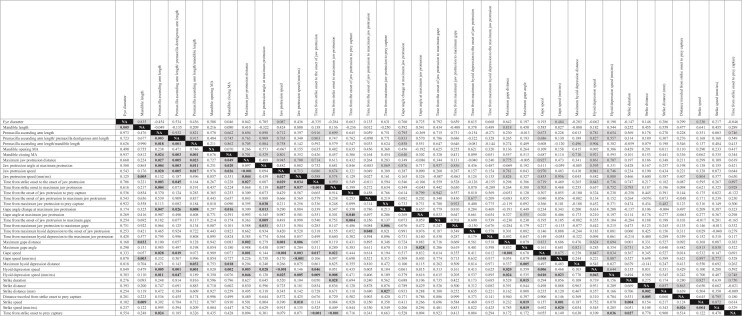	

Correlation coefficients above the diagonal; *P*-values below the diagonal. Significant *P*-values in bold and shaded (α = 0.05) with the corresponding correlation coefficients shaded.

The evolution of seven variables was significantly correlated with at least seven other variables each (Fig. 4, Table 4): premaxilla ascending arm length (scaled by fish standard length, SL; 9 sig. correlations), maximum jaw-protrusion distance (7 sig. correlations), premaxilla ascending arm length (scaled by dentigerous arm length; 7 sig. correlations), jaw-protrusion speed (8 sig. correlations), gape speed (7 sig. correlations), hyoid depression speed (8 sig. correlations), and mandible closing MA (7 sig. correlations). All 7 of these underwent correlated evolution with each other, except for gape speed and mandible closing MA (Fig. 4, Table 4). Because two of them were associated with the length of the ascending arm of the premaxilla, we collapsed them into one variable (premaxilla ascending arm length) in Fig. 4B.

### Giant danio exhibits differences in lower jaw length and aspects of feeding movements

The pANOVA testing revealed significant differences among body size classes in five variables: mandible length, distance traveled from strike onset to prey capture, strike speed, maximum gape distance, and gape speed (Table 2). For most of these variables, significance was maintained across the different body size groupings (see Table 2 for exceptions). *Post hoc* testing showed that only the giant danio (*Devario aequipinnatus*) was significantly different from other species for these five variables (Table 2). There were no significant differences for any of the variables between the environmental groups (water flow speeds).

The giant danio was the largest species examined by far (Table 2) and in all three size grouping schemes it was the sole occupant of the largest size class (either “large” or “extra large”). For mandible length, strike speed, and gape speed the giant danio was significantly different from the members of the next largest size class (Table 2). When using the second size classification scheme (“size 2”) the giant danio was significantly different from the next two largest size classes (Table 2). A similar pattern was also seen for the distance traveled from strike onset to prey capture, except when using the second size classification scheme. In this case the occupants of the “large” category (*D. albolineatus* and *D. feegradei*) were not found to be significantly different from the giant danio (“extra large”), but the giant danio was significantly different from those species in the “medium” category (Table 2). The giant danio was significantly different from those species in the smallest size classes in regard to maximum gape distance (Table 2).

### Rapid evolution of premaxilla anatomy and kinematics in Danio

**Fig. 5:**
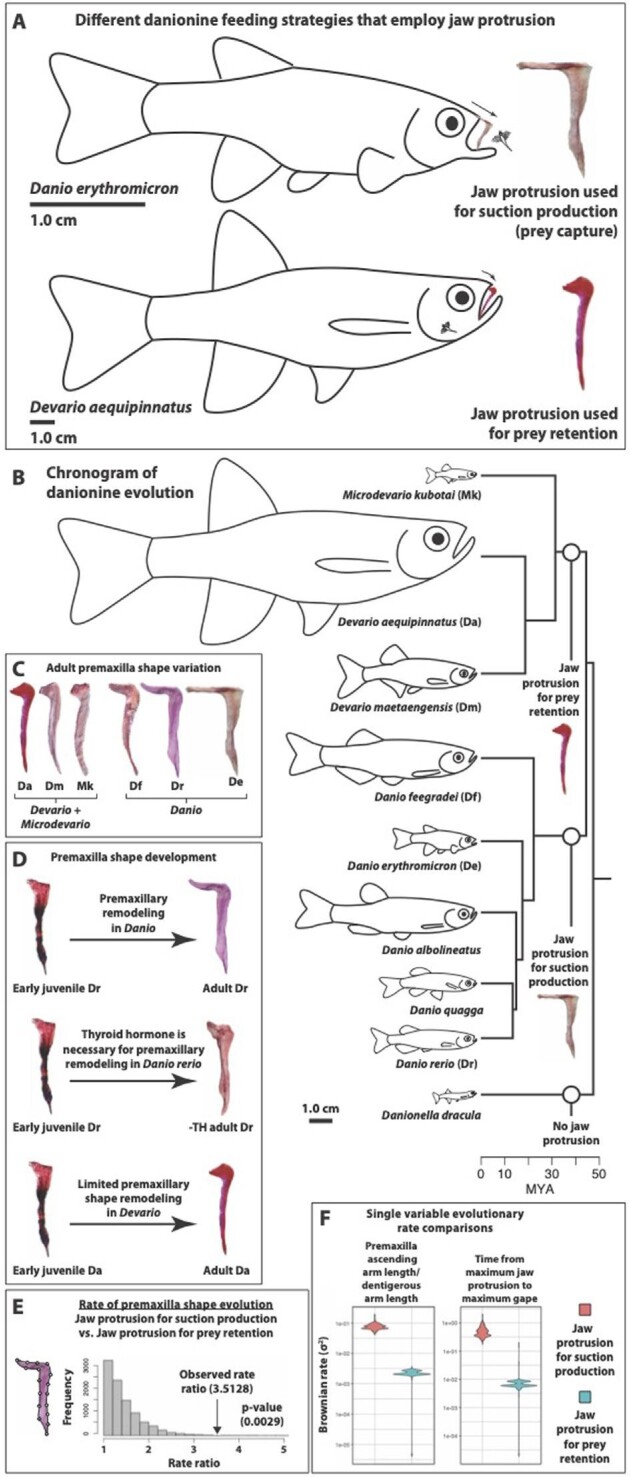
The evolution and development of danionine feeding mechanics. A. Tracings of two danionine species taken from high-speed videos at ∼80–85% maximum jaw protrusion. They typify two feeding strategies that employ alternative jaw-protrusion techniques and different premaxilla shapes: *Devario aequipinnatus* (typical of *Devario* and *Microdevario* species); *Danio erythromicron* (typical of *Danio* species). B. Chronogram showing the relationships and divergence times of the 9 species examined here. Body sizes are relative. The emergence of three feeding strategies associated with differences in jaw protrusion are marked. C. Variation in premaxilla shape among the Danioninae. There is limited variation in ascending arm length (all very short) in the DM clade. Ascending arms are longer in the *Danio* clade and there is greater length variation. D. Premaxilla development in the Danioninae. The ascending arm elongates during post-metamorphic development in wild-type zebrafish when thyroid hormone (TH) is present. Ascending arms do not elongate in hypothyroid zebrafish (-TH; Galindo et al. 2019). Post-metamorphic ascending arm elongation does not occur in *Devario aequipinnatus*. E. Mandible elongation in zebrafish mutants with congenitally elevated (+TH) levels. F. Changes in the ratio of premaxilla ascending arm length to dentigerous arm length evolved significantly faster among fishes that use jaw protrusion for suction production (*Danio*) than among those that use it for prey retention (DM). Changes in the relative timing of maximum jaw protrusion and maximum gape evolved significantly faster among fishes that use jaw protrusion for suction production (*Danio*) than among those that use it for prey retention (DM).

We observed significant differences in rates of premaxilla shape evolution between species that employed jaw protrusion earlier in a feeding strike when protrusion appeared to contribute to suction production and prey capture (*Danio*) and those that protruded their jaws significantly later and well after prey capture (DM; Fig. 5E, F). Those species with early jaw protrusion tended toward a faster rate of overall premaxilla shape evolution (σ^2^ = 1.51 × 10^−5^) relative to those with delayed jaw protrusion (σ^2^ = 4.29 × 10^−6^; *P* = 0.003; Fig. 5E). Similarly, we found faster rates of premaxilla ascending arm length evolution in “early protrusion” species (σ^2^ = 7.67 × 10^−2^) relative to “late protrusion” species (σ^2^ = 2.13 × 10^−3^; *P* = 0.011; Fig. 5F). The timing of maximum jaw protrusion relative to maximum gape also evolved faster in early protrusion species (σ^2^ = 4.69 × 10^−1^) relative to late protrusion species (σ^2^ = 6.06 × 10^−3^; *P* = 0.018; Fig. 5F).

## Discussion

### Jaw-protrusion strategy is linked to clade in the Danioninae

The diversification of danionine feeding mechanisms has been tightly linked to changes in jaw-protrusion mechanics. Alternate jaw-protrusion strategies are employed by each of the three major lineages (see supplementary data for video examples). Premaxilla shape, which in combination with the movement of the kinethmoid bone is a major determinant of protrusion ability (Staab and Hernandez 2005), is also distinct in each of these lineages (Figs. 2A and 5C). Of the 36 variables we examined (Table 1), only those associated with jaw protrusion showed significant differences between the *Danio* and DM clades (Table 3), and we saw no protrusion in *Danionella*. We found that premaxilla ascending arm length, jaw-protrusion distance, and jaw-protrusion speed have evolved in correlation with each other, and that these changes have been tightly integrated with the evolution of other highly important aspects of feeding mechanics: gape speed, hyoid depression speed, and the MA applied to the lower jaw during mouth closing (Fig. 4; Table 4).

*Danionella* do not, and likely cannot, use jaw protrusion. The *Danionella* clade, which is comprised exclusively of congeners, contains paedomorphic species that represent some of the world's smallest vertebrates (Roberts 1986; Britz et al. 2009; Tang et al. 2010; McCluskey and Postlethwait 2015; Stout et al. 2016; Britz and Conway 2016; Conway et al. 2020). *Danionella* have a feeding strike that is highly similar to that of larval zebrafish in which jaw protrusion has yet to develop (McMenamin et al. 2017). We saw no jaw protrusion in *Danionella dracula* (Figs. 2, 3, and 5), which may not possess the premaxillary bones necessary for this action (Britz et al. 2009), and this absence of jaw protrusion may be consistent throughout the genus. Extremely limited movement in the upper jaw region during *Danionella* feeding was reported previously, with the range of motion not significantly different from zero (McMenamin et al. 2017). While some rotation of upper jaw elements caused minor motion during feeding, there was no appreciable protrusion.

Although the *Danionella* lineage is basal within the Danioninae, an absence of jaw protrusion is not ancestral for the subfamily. Inspection of skull anatomy and feeding behavior in the two other danionid subfamilies (Chedrinae and Rasborinae *sensu*Stout et al. 2016; these subfamilies are synonymous with the tribes Chedrini and Rasborini *sensu*Tang et al. 2010) revealed fully ossified premaxillae in all species examined and the use of upper jaw protrusion in both lineages (data not shown). The functional morphology of *Danionella* feeding therefore appears to be both highly derived and consistent with the paedomorphic retention of larval feeding mechanisms (McMenamin et al. 2017; Hu et al. 2019a).

The DM clade appears to utilize jaw protrusion for prey retention. Upper jaw movement began immediately before prey capture, but only very slight protrusion had occurred by the time *Artemia* had been drawn into the mouth (Fig. 3). Their premaxillae have short ascending arms (Figs. 2 and 5; Table 3), so that even full protrusion would not contribute to sufficient expansion of the buccal cavity to produce useful suction. Furthermore, their premaxillary shapes are convergent with those of damselfishes that employ only limited use of suction during feeding (Cooper et al. 2017).

Coordinated gape and hyoid depression were used in the suction-based capture of *Artemia* by all species examined here, including those in the genera DM (Fig. 3), but DM species employed jaw protrusion too late to allow any resulting suction, however slight, to combine with the other suction forces used for prey capture (Fig. 3). In comparison to their sister lineage (*Danio*; Fig. 5), DM species performed jaw protrusion significantly later in their feeding strikes relative to prey capture, maximum gape, and maximum hyoid depression (Table 3). Their mouths had also closed significantly more than those of *Danio* species before maximum protrusion was reached (Fig. 3, Table 3) and jaw protrusion was directed toward the lower jaw instead of parallel to it as in *Danio* (Fig. 5A, Table 3). In order to contribute to suction production, jaw protrusion must rapidly expand the volume of the buccal cavity (Wainwright et al. 2015), but protrusion toward the lower jaw at low gape angles would not accomplish this.

We interpret the use of jaw protrusion in DM as a mechanism of prey retention. Water drawn into the buccal cavity by suction feeding must be expelled, and if this expulsion is primarily in a posterior direction (through the opercular openings), then prey can be collected on gill rakers (Langeland and Nost 1995). The larger the mouth opening during buccal cavity compression, the greater the probability that captured prey will be expelled forward and lost. In all species examined here, the start of mouth closing preceded compression of the buccal cavity via hyoid abduction (Fig. 3), which would reduce the chances of captured prey being ejected from the mouth. Protruding the premaxillae toward the lower jaw at low gape angles would further constrict the mouth opening and reduce anterior water flow during buccal cavity compression. Although the direction, timing, and degree of jaw protrusion in DM are inconsistent with any contribution to suction production and prey capture, their use of jaw protrusion would direct water though the gill arches during buccal cavity compression and therefore enhance prey retention. Examination of other genera in the DM lineage (e.g., *Chela, Inlecypris, Laubuka, Microrasbora, Neochela*; Tang et al. 2010; Stout et al. 2016) would permit the determination of whether this feeding strategy is a synapomorphy of the clade.

*Danio* species use jaw protrusion for suction-based prey capture. We observed *Artemia* accelerating toward the mouth as *Danio* approached full jaw protrusion (Fig. 5A) and maximum protrusion and maximum hyoid depression were nearly simultaneous, which would allow the forces generated by these motions to have additive effects on suction production (Fig. 3). The direction of jaw protrusion was more parallel with the lower jaw in *Danio* relative to DM, so this action would contribute to expansion of the buccal cavity (and therefore contribute to suction production) rather than closing off its anterior opening (Fig. 5A, Table 3). The long ascending arms of *Danio* premaxillae are also found in damselfish species that specialize in suction feeding (Cooper et al. 2017).

The greater degree of premaxilla shape variation among *Danio* species (Fig. 5C) is consistent with higher evolutionary rates of premaxilla form and function relative to the DM clade (Fig. 5E, F). The tight connection between premaxilla shape and diet seen in other species (Cooper et al. 2017) suggests that trophic diversity may also be higher in *Danio*. Phylogenetic analyses of form, function, and feeding ecology have provided valuable insight into the diversification of many fish radiations, particularly reef fishes (e.g., Westneat et al. 2005; Konow et al. 2008; Cooper and Westneat 2009; Cooper et al. 2017). Additional diet data for a larger number of danionin species would provide a useful complement to studies of their trophic form and function.

Because species sampling was limited in this study additional work on danionine feeding mechanics is necessary before a reliable picture of their trophic evolution can emerge. Broader taxonomic sampling of *Danio, Danionella*, and DM species would allow for more robust statistical analyses that could test the validity of the findings reported here. The feasibility of this expansion is supported by the commercial availability of many danionine species and the ease with which they may be maintained in aquaria.

### Trophic modularity in the Danioninae

We found strong evidence for modularity in the functional morphology of danionine feeding. Premaxilla ascending arm length, jaw-protrusion speed, jaw-protrusion distance, gape speed, hyoid depression speed, and mandible closing MA represent an evolutionary module because these traits have changed in a tightly correlated manner during danionine diversification (Fig. 4, Table 4; Wagner and Altenberg 1996; Wagner et al. 2007). They also constitute a clear functional module because they act together during feeding (West-Eberhard 2003; Westneat 2004; Wagner et al. 2007).

Although mandible closing MA is part of this module, mandible opening MA is not, nor has mandible opening MA undergone correlated evolution with any of the other traits we examined in the Danioninae. Evans et al. (2019) found a significant relationship between functional performance and mandible closing MA in navajine electric fishes, but no significant connection between mandible opening MA and functional performance. Evans et al. (2019) attributed this finding to the many-to-one mapping of morphological traits to functional performance, but it is also possible that the calculation of mandible opening MA used both in this study and by Evans et al. (2019) does not adequately capture important aspect of jaw opening mechanics. The protractor hyoidei muscle, for example, inserts near the anterior ends of the dentary bones (Winterbottom 1973) and can play an important role in abducting fish mandibles (Stiassny 2000). This muscle is also better positioned to apply a higher MA during fish mouth opening than is the interopercular ligament (Winterbottom 1973). This ligament's attachment to the posterior tip of the angular (the retroarticular of some authors) is frequently used in calculations of fish mandible opening MA (e.g., Fig. 1; Cooper and Westneat 2009; Baumgart and Anderson 2018; Evans et al. 2019), but its role in mouth opening many have been over emphasized.

The anatomical elements which contribute to jaw protrusion, gape, hyoid depression, and mouth closing in the Danioninae are likely to be strong determinants of trophic niche (Cooper et al. 2017). Selection for efficient feeding may therefore have contributed their having evolved in a correlated manner. It is also possible that this pattern is the product of shared mechanisms of morphogenesis (Klingenberg 2008). Corresponding patterns of genetic, developmental, and functional integration and their role in the adaptive diversification of feeding have been studied extensively in fishes, especially in rift-lake cichlids (e.g., Cooper et al. 2011; Parsons et al. 2012, 2014; Powder et al. 2015; Hu and Albertson 2017; Ahi et al. 2019; Conith et al. 2020). There have also been multiple studies of integration and/or modularity in the zebrafish skull (Stock 2001; Depew and Simpson 2006; Fish et al. 2011; Huycke et al. 2012; Kimmel 2014; Lehoux and Cloutier 2015; Parsons et al. 2018), but in light of the fact that this species is used extensively to understand the genetic controls of craniofacial development, such studies are not as numerous as might be expected.

An investigation of modularity that examined the skull morphology of wild-caught zebrafish (obtained from the Kosi River, India, 2015) did not find that any aspects of premaxilla morphology belonged to the best-supported cranial modules. This was also true of specimens from the AB wild-type laboratory strain (the same as was examined here), but premaxillae from fish of the Tübingen wild-type strain showed strong integration with the maxilla and bones in the suspensorium (Parsons et al. 2018). Functional integration that allows successful feeding has obviously been retained in all three lines. It is therefore possible that the functional integration we see in the evolution of danionine trophic morphology could be achieved using skulls with multiple patterns of shape integration among cranial elements. Testing for different patterns of modularity in skull shape among danionine species could determine whether shifts in cranial modularity have accompanied changes in feeding mechanics.

### Hormonal and heterochronic changes can shift feeding mechanics in Danioninae

Like many fishes, zebrafish begin significant skull remodeling during metamorphosis and some aspects of this restructuring continue into juvenile and even adult development (Galindo et al. 2019; Nguyen et al. 2022). Due to the high water viscosities they experience, using jaw protrusion for suction feeding would be difficult for pre-metamorphic (i.e., larval) danionines, and possibly for adult *Danionella* since they are not much larger, because this motion would be more likely to push prey forward than to draw it into the mouth (Galindo et al. 2019). Zebrafish do not develop jaw protrusion until after a spike in thyroid hormone (TH) blood levels triggers their metamorphosis (McMenamin and Parichy 2013; Galindo et al. 2019).

Transgenic zebrafish rendered incapable of producing TH develop ossified premaxillae in which the ascending arms do not elongate (Fig. 5D; Galindo et al. 2019). The adult premaxillae of these hypothyroid specimens closely resemble those of juvenile wild-type zebrafish, juvenile *Devario aequipinnatus*, and adult DM (Fig. 5D). Zebrafish mutants that produce excess TH (i.e., there are hyperthyroid) develop elongated mandibles (Fig. 5E; Galindo et al. 2019). The mandibles of *Devario aequipinnatus* are significantly longer than those in the other danionines we examined (Table 2) and many of the giant danio we reared had mandibles that projected beyond their upper jaws (i.e., they were prognathous), which is typical of hyperthyroid zebrafish (Fig. 5E; McMenamin et al. 2017; Galindo et al. 2019). The larger gape distances and faster gape speeds of giant danio may, at least in part, be the product of their longer lower jaws because increased mandible length would increase both variables even if gape angles remained constant (Table 2). Changes in mandible length have the potential to shift fishes into different feeding niches, and elongated mandibles can contribute to the superior (i.e., upturned) mouth position seen in many species that feed from below (e.g., surface feeders; Helfman et al. 2009).

TH signaling can have highly disparate effects in different tissues and organs, including different bones or skeletal regions (Berry et al. 1998; Bassett and Williams 2016; Keer et al. 2019, 2022; Hu et al. 2019b). The evolution of divergent responses to TH in the upper and lower jaws of the same species is therefore highly plausible. *Devario aequipinnatus* represents an interesting comparison to zebrafish, because they develop upper jaw elements (premaxillae) similar to those in hypothyroid zebrafish, and lower jaws (mandibles) similar to those in hyperthyroid zebrafish (Fig. 5D, E).

In a large number of fish species, important aspects of adult trophic morphology arise during the cranial remodeling that begins with metamorphosis (McMenamin and Parichy 2013; Cooper et al. 2020). Evolutionary changes to how cranial elements respond to TH therefore have the potential to produce adults with different feeding mechanics (Shkil et al. 2012; McMenamin et al. 2017; Galindo et al. 2019; Keer et al. 2019, 2022). Because it acts to coordinate changes in multiple skeletal elements (Shkil et al. 2012; Leitch et al. 2020), patterns of modularity in adult fish skulls may be strongly affected by changes in how different bones or regions respond to TH. Multiple studies have recently suggested that changes in TH signaling have played an important role during the trophic diversification of fishes (Shkil et al. 2012, 2015; Galindo et al. 2019; Cooper et al. 2020) and this may have been the case with the species examined here.

### Jaw protrusion as a key innovation and target for Evo–Devo

Jaw protrusion is one of the most important key innovations to have evolved in vertebrates. Its adaptive value is underscored by the fact that it has evolved independently at least seven times via a range of mechanisms, and that approximately one-third of living vertebrates (>21,000 species, all fishes) belong to lineages in which jaw protrusion is an ancestral trait (Wilga et al. 2007; Wainwright et al. 2015). The large majority of these species are members of just two lineages of the Actinopterygii (ray-finned fishes): the Acanthopterygii (spiny-rayed fishes, ∼17,000 species) and the Cypriniformes (minnows and their close relatives, ∼3200 species; Wainwright et al. 2015). In both clades the length of the ascending arm of the premaxilla can be an important determinant of protrusion ability (Fig. 1; Cooper et al. 2017).

Part of the utility of jaw protrusion stems from the fact that it can be used in a variety of ways. In damselfishes (Pomacentridae; Acanthopterygii), protrusion ability is tightly associated with trophic niche, with highly protrusile species using suction to capture zooplankton in the water column (Cooper et al. 2017). Among the Cyprinidae (minnows, carps), which are very closely related to the Danionidae (Tang et al. 2010; Stout et al. 2016), protrusile jaws are frequently used to suck invertebrates or decaying plant matter from sediments (Sibbing et al. 1986; Sibbing 1988; Paszkowski et al. 1996).

Protrusile jaws are not always used for suction feeding. Many species of squamipinne fishes (Acanthopterygii, Squamipinnes; marine angelfishes, butterflyfishes, surgeonfishes, and their relatives) have biomechanical arrangements that allow them to bite with their jaws protruded (Konow et al. 2008), which promotes their ability to feed on sponges, tunicates, and other tough invertebrates attached to hard substrates (Konow et al. 2008; Konow and Bellwood 2011). Parrotfishes (Acanthopterygii, Labridae, Scarinae) use protrusile jaws to scrape algae or other material from rocks and coral heads, or to feed on seagrasses (Rice and Westneat 2005).

Another advantage stems from the evolvability of acanthomorph and cypriniform jaw-protrusion mechanisms. When only slight changes to the functional morphology of feeding can have large ecological consequences, the basic biomechanical arrangement represents a highly evolvable structure. Even slight changes in the length of the premaxillary ascending arm and the timing at which protrusion occurs can shift danionines between different feeding strategies, and in damselfishes changes in ascending arm length are a major determinant of whether fishes feed from the water column or the benthos (Cooper et al. 2017). The ascending arm of the premaxilla is anatomically simple, and variation in its length is likely to exist within populations of fishes that possess this feature. Such variation would promote their ability to shift between food sources as environments change or when they invade new habitats.

Because small changes to protrusion mechanisms can alter feeding ecology, and because jaw protrusion is associated with extraordinary evolutionary success, the developmental determinants of protrusion ability represent an attractive target for Evo–Devo. TH signaling may be a productive area for such work. It is the trigger for fish metamorphosis (before which jaw protrusion is not known to exist), is necessary for premaxillary ascending arm elongation in zebrafish, plays an important role in regulating bone development, and has the potential to integrate the development of multiple skull regions (McMenamin and Parichy 2013; Bassett and Williams 2016; McMenamin et al. 2017; Galindo et al. 2019; Keer et al. 2019, 2022; Nguyen et al. 2022). An understanding of why the premaxillae of adult *Devario*, for example, resemble those of *Danio* that do not produce TH could help explain why giant danios don't suck nearly as much as zebrafish.

## Supplementary Material

obac049_Supplemental_Videos

## Data Availability

The data underlying this article and the R-scripts used to analyze those data will be shared on reasonable request to the corresponding author.
